# Effects of Low-Temperature Stress and Brassinolide Application on the Photosynthesis and Leaf Structure of Tung Tree Seedlings

**DOI:** 10.3389/fpls.2019.01767

**Published:** 2020-01-31

**Authors:** Fanhang Zhang, Kun Lu, Yiyang Gu, Lin Zhang, Wenying Li, Ze Li

**Affiliations:** ^1^ Key Laboratory of Cultivation and Protection for Non-Wood Forest Trees, Ministry of Education, Central South University of Forestry and Technology, Changsha, China; ^2^ College of Food Science, Central South University of Forestry and Technology, Changsha, China

**Keywords:** *Vernicia fordii*, *Vernicia montana*, low-temperature stress, brassinolide, chloroplast ultrastructure, chlorophyll fluorescence

## Abstract

The tung tree is an important woody oil tree species. Tung oil extracted from the tung fruit seeds is used in the manufacture of environmentally friendly paint. This study investigated the effects of the application of brassinolide (BR) under different temperature conditions on the chlorophyll content, photosynthesis, chlorophyll fluorescence, leaf structure, and chloroplast ultrastructure in *Vernicia fordii* and *Vernicia montana*. The conditions used were 8°C-Control (low temperature and no BR), 8°C-BR (low temperature and BR application), 28°C-Control (normal temperature and no BR), and 28°C-BR (normal temperature and BR application), and effects were monitored from 5 to 15 days after the treatments (DAT). The results showed that the low temperature treatment (8°C-Control) significantly reduced the net photosynthetic rate (*P_n_*), stomatal conductance (*G_s_*), maximum fluorescence (*F_m_*), maximum photochemical efficiency (*F*_v_/*F*_m_), and actual photochemical and quantum efficiency (*Φ*_*PSII*_) compared to the control condition (28*°C-Control)*. However, the external application of BR alleviated the negative effects of low-temperature stress to some degree for all the above parameters for both species tested, except for *P*_n_ and *G*_*s*_ at 15 DAT. There were no significant differences in most of the parameters in either species between the 28°C-Control and 28°C-BR treatments. At 10 and 15 DAT of low-temperature stress, the 8°C-Control treatment significantly reduced leaf cell tense ratio (CTR) and increased spongy ratio (SR) compared to the 28°C-Control, whereas BR application alleviated the adverse effects. Moreover, the 8°C-Control treatment significantly destroyed the chloroplast structure, loosening the thylakoids until they disintegrated, while exogenous spraying of BR protected the chloroplast structure and enabled it to function properly in both species. Our results suggested that long-term low temperatures significantly reduced the photosynthetic efficiency of tung tree seedlings, affecting the formation of the internal structure of plant leaves and destroying the integrity and function of the chloroplast. To prevent this, external application of BR to tung tree seedlings could enhance the photosynthetic potential of tung trees by maintaining the stability of the leaf structure, morphology, and function, and alleviating the damage caused by cold injury. The results also showed that *V. fordii* seedlings are more resistant to low temperatures than *V. montana* seedlings.

## Introduction

The tung tree is a heliophile that grows quickly, yielding fruits within three years due to its high photosynthetic efficiency (Shockey et al., 2006; Chen et al., 2019). *Vernicia fordii* is a deciduous tree belonging to the Euphorbiaceae. Native to China, it is one of the four woody oil tree species in China with a long history of cultivation and high economic value (Gu et al., 2019; Liu et al., 2019; Zhang et al., 2019). *Vernicia montana* is also a deciduous tree belonging to the Euphorbiaceae. It is tall and strong and has excellent disease resistance and a longer fruiting period than *V. fordii *(Li et al., 2017a; Liu et al., 2019a). Tung oil is a widely used material in environmental protection coatings. It has excellent adhesion and glossiness, is fast-drying, and is resistant to heat, acids, alkalis, and cracking as a result of frost. Additionally, tung oil used in environmental paints is harmless to humans and therefore has a broad range of applications (Cao and Shockey, 2012; Li et al., 2017a; Li et al., 2019). Previous research has shown that the tung tree is one of the most promising biomass energy tree species (Tan et al., 2011; Lin et al., 2016).

Temperature is a major environmental factor that limits the distribution, productivity, and survivability of plants (Theocharis et al., 2012). Wu et al. (2015) confirmed that the expansion of tea plant (*Camellia sinensis*) cultivation is restricted due to temperature. Low-temperature conditions can damage the apple epidermis, causing serious losses in productivity (Rudell et al., 2011). In recent years, along with global climate changes, the frequency, duration, and severity of low-temperature episodes have increased in many regions of the world (Kodra et al., 2011; Sanghera et al., 2011). Plants experiencing low-temperature and freeze injury often exhibit serious physiological and morphological responses, including damage to various cellular structures and decreased chlorophyll content, photosynthetic rate, stomatal conductance, transpiration rate, and electron transport rate (ETR) (Moon et al., 1995; Saxby et al., 2003; Ogweno et al., 2009; Schreiber et al., 2013). However, plants can use different strategies and undergo physiological reactions to adapt to low-temperature conditions. For example, endogenous ABA and ascorbic acid concentrations in the leaves and shoots increase in response to the low-temperature damage (Dallaire et al., 1994; Yoshikawa et al., 2007; Stevens et al., 2008). Furthermore, low temperature conditions cause a decrease in the chlorophyll content, superoxide dismutase activity, and maximal quantum yield of PSII photochemistry but an increase in dark respiration and abscisic acid content (Huner et al., 1993; Moon et al., 1995; Yoshikawa et al., 2007; Ogweno et al., 2009).

The primary problem of freezing injury is reductions in yield due to the killing of tung trees in the north subtropical zone (Chen, 1987; He and Wu, 1991). He and Wu (1991) revealed that the low-temperature condition seriously affected the germination and growth of the tung tree. More recently, in China, cold snaps have occurred more frequently in spring, leading to serious damage to the growth and development of tung trees and resulting in large economic losses (Zhang et al., 2018). In addition, a low-temperature environment causes a reduced seed number and seed oil content in *Jatropha curcas* L., which belongs to Euphorbiaceae and has growth habits similar to those of the tung tree (Xiong et al., 2011; Wang et al., 2013). To date, no reports exist on the effects on photosynthesis and chloroplast ultrastructure of tung trees under different temperature treatments. It is vital to investigate cold resistance in tung trees and to find an effective means to alleviate the damage caused by cold weather.

Brassinolide (BR) is an important plant stress hormone that regulates physiological processes and can promote plant resistance to low-temperature environments (Shakirova et al., 2016; Wani et al., 2017; Vardhini, 2017; Chen et al., 2018). By analyzing the expression of a subset of cold-stress marker genes, Kagale et al. (2006) confirmed that BR treatment enhanced the tolerance of *Arabidopsis thaliana* and *Brassica napus* to cold stress. Moreover, BR application in cases of cold stress increased the chlorophyll content of *Cucumis sativus* L., enhanced photosystem II and antioxidant enzymes activity, and protected the photosynthetic membrane system from oxidative damage (Fariduddin et al., 2011). Fujii and Saka (2001) confirmed that BR promoted cell elongation in young rice seedlings under low-temperature stress. Vardhini (2017) reported that anatomical characteristics of plants could be ameliorated and protected by BR application under various abiotic stress conditions. Therefore, it is important to explore the mechanisms of BR to improve the low-temperature tolerance of tung tree seedlings, especially in terms of photosynthesis, anatomical structure, and chloroplast ultrastructure.

We studied the effects of exogenous BR on the photosynthetic characteristics, leaf anatomical structure, and chloroplast ultrastructure of two species of tung tree seedlings under different temperature conditions. Our objective was to explore the mechanisms underlying the BR-induced alleviation of long-term low-temperature stress on the photosynthetic processes of the two tung trees species. The chlorophyll content, photosynthesis, and chlorophyll fluorescence of the tung tree species were measured, and the leaf anatomical structure and chloroplast ultrastructure were investigated.

## Materials and Methods

### Plant Materials and Treatments

Seedlings of *V. fordii* and *V. montana* were obtained from the national conservation bank of tung tree germplasm resources (Xiangxi Region, Hunan Province, China). On January 15, 2018, seeds were sterilized in 0.5% KMnO_4_ for 20 min. The sterilized seeds were stored in wet sand for 3 months, and germinated seeds were selected and transplanted on March 30, 2018 to rounded plastic containers (inner diameter, 25 cm; height, 20 cm) containing red soil, perlite, and vermiculite at a ratio of 1:1:1 (v/v/v). The plants were grown under natural conditions at the Central South University of Forestry and Technology, Changsha, China (28°10' N; 113°23' E).

From June 8, 2018 to June 10, 2018, BR (0.05 mg·L^-1^, 15 mL per pot) was sprayed on the tung tree seedlings at 7:00 h, and the same dose of pure water was sprayed on other seedlings as a control. In the morning of June 11, 2018, all plant materials were transferred to a controlled growth chamber at day/night low temperatures of 8/5°C and day/night normal temperatures of 28/25°C, with 70% relative humidity and a 12h d^-1^ photoperiod. The lamp parameters were 16W, 220V, 50Hz, 9×, and a photosynthetic photon flux density (PPFD) of 200 µmol·m^-2^·s^-1^. Each treatment contained 18 pots of seedlings, and the whole experiment comprised 144 pots of seedlings.

The soil water content was maintained at an 80 ± 5% field capacity (FC) level by watering each day during the experiment. The photosynthetic pigment content, photosynthetic efficiency, and chlorophyll fluorescence parameters of the tung tree seedlings were measured on June 15 (5 days after treatment [5 DAT]), June 20 (10 DAT), and June 25 (15 DAT). Samples were also taken for observing leaf anatomy structure on these three days, and samples for observing chloroplast ultrastructure were taken on 15 DAT.

### Chlorophyll Content

Leaves at a similar position were selected and cut into very thin filaments. The filaments were soaked in an acetone-ethanol mixture (1:1, v/v) for 24 h in 4°C and darkness until they turned white. The filaments were shaken evenly every 2 h during the experiment. The absorbance at 663 and 645 nm in the solution was measured with a spectrophotometer (UV-1100, Mapada, China), and the chlorophyll a (Chl a) and b (Chl b) concentrations were calculated: C (Chl a) (mg·dm^-2^) = (12.72× A_663_)–(2.59× A_645_), C (Chl b) (mg·dm^-2^) = (22.88× A^645^)–(4.68× A^663^), C (Chla+b)(mg·dm^-2^) = C (Chl a) + C (Chl b). All chlorophyll content data in this work were presented as average ± standard error (SE) of three biological replicates.

### Photosynthetic Physiological Parameters

Photosynthetic physiological parameters were measured between 9:00 and 11:00 h (one leaf per plant; three plants per replicate) using a LI-6400XT Portable Photosynthesis System (LI-COR, Lincoln, NE, USA). The net photosynthetic rate (*P*_*n*_), stomatal conductance (*G*_*s*_), intercellular CO_2_ concentration (*C_i_*), and transpiration rate (*T*_*r*_) were measured at a PPFD source of 1,000 µmol·m^-2^·s^-1^ provided by LEDs emitting blue- and red-light sources. The leaf measurement area was 6 cm^2^ and the air flow rate was 500 µmol·s^-1^. The instantaneous water-use efficiency (WUE) was calculated as WUE = *P*_*n*_/ *T*_*r*_ (Qu et al., 2019). CO^2^ was supplied at a concentration of 400 µmol·mol^-1^ using small cylinders. The temperature in the leaf cuvette under normal/low-temperature conditions was 28/8°C, and the relative humidity was 70%. All photosynthetic data in this work were presented as average ± SE of three biological replicates.

### Chlorophyll Fluorescence Parameters

Complete portable fluorescent-photosynthetic gas exchange assay system (LI-COR, USA) was connected by the 6400-40 fluorescent leaf chamber and the LI-6400XT analyzer. The system was used to measure chlorophyll fluorescence parameters. Mature leaves were selected and marked before the first measurements were taken. The chlorophyll fluorescence parameters of the labelled leaves were determined after a 2-h dark treatment between 21:00 and 23:00 and after 60 min of photo activation between 9:00 and 10:00. The maximum fluorescence (*F*_*m*_), maximum photochemical efficiency (*F*_*v*_ */F*_*m*_), actual photochemistry quantum efficiency (*Φ*_*PSII*_), and photochemistry quenching coefficient (*qP*) were measured. The air flow rate was 300 µmol·s^-1^, and the leaf measurement area was 2 cm^2^. All chlorophyll fluorescence data in this work were presented as average ± SE of three biological replicates.

### Leaf Anatomical Structure

The leaf anatomical structure was analyzed by imaging leaf sections using an optical microscope. The third functionally mature leaf below the shoot tip of seedlings was selected, and 5- × 4-mm pieces were cut from the middle of the leaf near the vein. Three samples from each treatment group were chosen. The samples were fixed in formaldehyde alcohol acetic acid solution (FAA, 1:1 [v/v]) for 24 h and then placed in 70% ethanol and stored at 4°C. The samples were dehydrated in a series of graded ethanol solutions (70%, 85%, 90%, 95%, and 100%), infiltrated with paraffin wax for polymerization, sectioned (three pieces per sample) using a microtome (Leica, GER), dyed (leaves of *V. fordii* were dyed with saffron and Fast Green, and leaves of *V. montana* were dyed with saffron), and imaged under a microscope (Leica Gre, Heidelberg, Germany). The best and most representative image of each treatment group was selected to reveal (**Figures 6** and **7**). The palisade tissue, spongy tissue, and total thickness of leaves were measured. The leaf cell tense ratio (CTR) was calculated as CTR = palisade tissue/total leaf thickness. The leaf spongy ratio (SR) was calculated as SR = sponge tissue/total leaf thickness. All measured data were presented as average ± SD of three biological replicates.

### Chloroplast Ultrastructure Observation

The middle portion of each leaf blade was cut into 1-mm^2^ strips and fixed in 2.5% glutaraldehyde solution (prepared with 0.1 mol L^–1^ sodium phosphate, pH 7.3) for 24 h at 4°C. After washing three times (30 min each), the tissue was fixed in 1% osmium tetroxide for 2 h at room temperature and dehydrated in a series of graded ethanol solutions (Gao et al., 2015). The leaf samples were then embedded with epoxy resin, placed in an ion sputter coater, and gilded for 20 min. Semi-thin sections (0.5 µm) were cut with a diamond knife using an ultramicrotome (EM UC7, Leica) and mounted on copper grids. Imaging was performed using a transmission electron microscope (HT7700; Hitachi, Tokyo, Japan). We selected two representative images from all of our obtained images of each treatment group (**Figures 8** and **9**).

### Statistical Analysis

Microsoft Office Excel 2013 was used to process the data. Origin 9.0 was used to create the plots, and SPSS 19.0 software was used to analyze the variance to test for differences. Treatment means were compared using one-way analysis of variance (ANOVA) and Duncan's multiple range test with a probability of *p* ≤ 0.05.

## Results

### Chlorophyll Content

**Figure 1:**
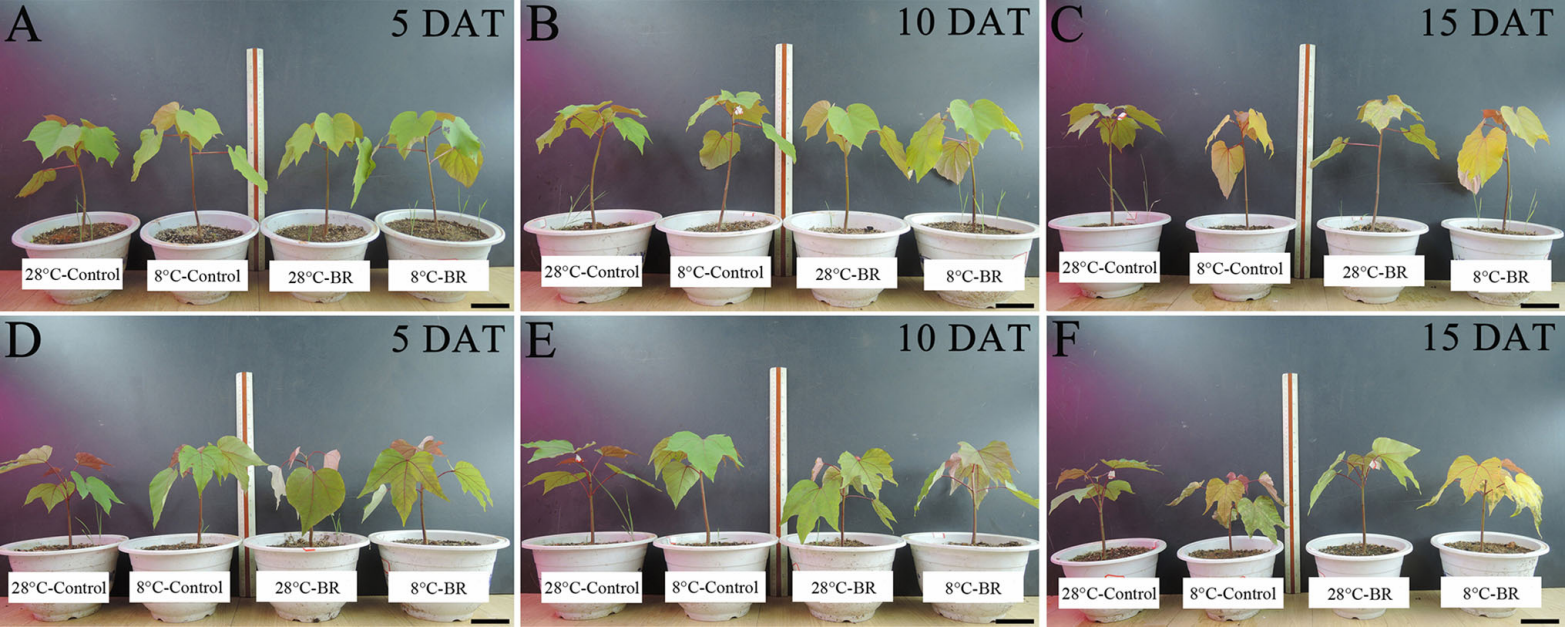
Effects of BR on the growth of two tung tree species under normal and low-temperature conditions. Treatments were (1) 28°C-Control, normal temperature and no BR; (2) 28°C-BR, normal temperature and BR application; (3) 8°C-Control, low temperature and no BR; and (4) 8°C-BR, low temperature and BR application. Species were (1) Vernicia fordii-**(A–C)** (2) *Vernicia montana*-**(D–F)** Scale bars are 10 cm.

With prolonged low-temperature stress, the leaves of both species gradually became dehydrated, withered, and yellow (**Figure 1**). Compared with the 28°C-Control, the Chl a, Chl b, and Chl a+b contents of both species were significantly reduced under low temperature at 10 and 15 DAT, but the 8°C-Control treatment resulted in increased Chl a, Chl b, and Chl a+b contents at 5 DAT (**Table 1**). At the normal temperature of 28°C, exogenous spraying of BR resulted in increased Chl a, Chl b, and Chl contents in both species compared to the 28°C-Control. Similar results were achieved for both species with BR spraying at the low temperature, though with an exception at 15 DAT (**Table 1**).

**Table 1:** Effects of BR on chlorophyll content of two tung tree species under normal and low-temperature conditions.

Species	Treatment time/DAT	Treatment	Chl a content (mg·dm^-2^)	Chl b content (mg·dm^-2^)	Chl a+b content (mg·dm^-2^)
*Vernicia fordii*	5	8°C-Control	1.56 ± 0.08Aa	0.64 ± 0.06Aa	2.31 ± 0.12Aa
		8°C-BR	1.60 ± 0.03Aa	0.71 ± 0.01Aa	2.34 ± 0.08Aa
		28°C-Control	1.07 ± 0.02Bb	0.45 ± 0.01Ba	1.53 ± 0.03Ba
		28°C-BR	1.19 ± 0.14Ba	0.52 ± 0.05Ba	1.71 ± 0.20Ba
	10	8°C-Control	0.64 ± 0.04Bb	0.24 ± 0.01BCb	0.91 ± 0.05Bb
		8°C-BR	0.70 ± 0.06Bb	0.30 ± 0.01Ab	1.05 ± 0.14ABb
		28°C-Control	0.86 ± 0.05Ac	0.26 ± 0.02Bb	1.03 ± 0.08ABb
		28°C-BR	0.89 ± 0.08Ab	0.27 ± 0.02ABb	1.15 ± 0.12Ab
	15	8°C-Control	0.55 ± 0.10Bb	0.22 ± 0.03Ab	0.68 ± 0.17Bb
		8°C-BR	0.22 ± 0.02Cb	0.09 ± 0.02Bc	0.29 ± 0.06Cc
		28°C-Control	1.20 ± 0.04Aa	0.28 ± 0.04Ab	1.54 ± 0.07Aa
		28°C-BR	1.29 ± 0.09Aa	0.29 ± 0.04Ab	1.66 ± 0.13Aa
*Vernicia montana*	5	8°C-Control	2.22 ± 0.19Aa	0.82 ± 0.08Ba	3.04 ± 0.27Aa
		8°C-BR	2.25 ± 0.09Aa	0.97 ± 0.08Aa	3.11 ± 0.11Aa
		28°C-Control	1.03 ± 0.04Bc	0.53 ± 0.06Ca	1.52 ± 0.04Bb
		28°C-BR	1.16 ± 0.27Bb	0.69 ± 0.07Ba	1.69 ± 0.37Bb
	10	8°C-Control	1.09 ± 0.05Cb	0.35 ± 0.02Cb	1.36 ± 0.19Cb
		8°C-BR	1.11 ± 0.03Cb	0.40 ± 0.01BCb	1.51 ± 0.03Cb
		28°C-Control	1.61 ± 0.10Bb	0.49 ± 0.06Ba	2.01 ± 0.18Ba
		28°C-BR	2.03 ± 0.14Aa	0.69 ± 0.08Aa	2.55 ± 0.20Aa
	15	8°C-Control	1.12 ± 0.20Bb	0.22 ± 0.02Cc	1.34 ± 0.18Cb
		8°C-BR	0.84 ± 0.01Cc	0.20 ± 0.02Cc	1.06 ± 0.02Dc
		28°C-Control	1.96 ± 0.06Aa	0.31 ± 0.04Bb	2.14 ± 0.15Ba
		28°C-BR	2.11 ± 0.09Aa	0.64 ± 0.04Aa	2.62 ± 0.16Aa

Data represent mean values ± standard error (SE) (n = 3). Treatments were (1) 28°C-Control, normal temperature and no BR; (2) 28°C-BR, normal temperature and BR application; (3) 8°C-Control, low temperature and no BR; and (4) 8°C-BR, low temperature and BR application. Different uppercase/lowercase letters indicate significant differences at different treatments and times, indicated by p ≤ 0.05 according to Duncan's multiple range tests.

The Chl content of both species under low-temperature stress at 10 and 15 DAT was significantly lower than that of the control at 5 DAT (**Table 1**). Exogenous spraying of BR appeared to ameliorate the reduction in Chl content at 5 and 10 DAT (**Table 1**).

### Photosynthetic Parameters

**Figure 2:**
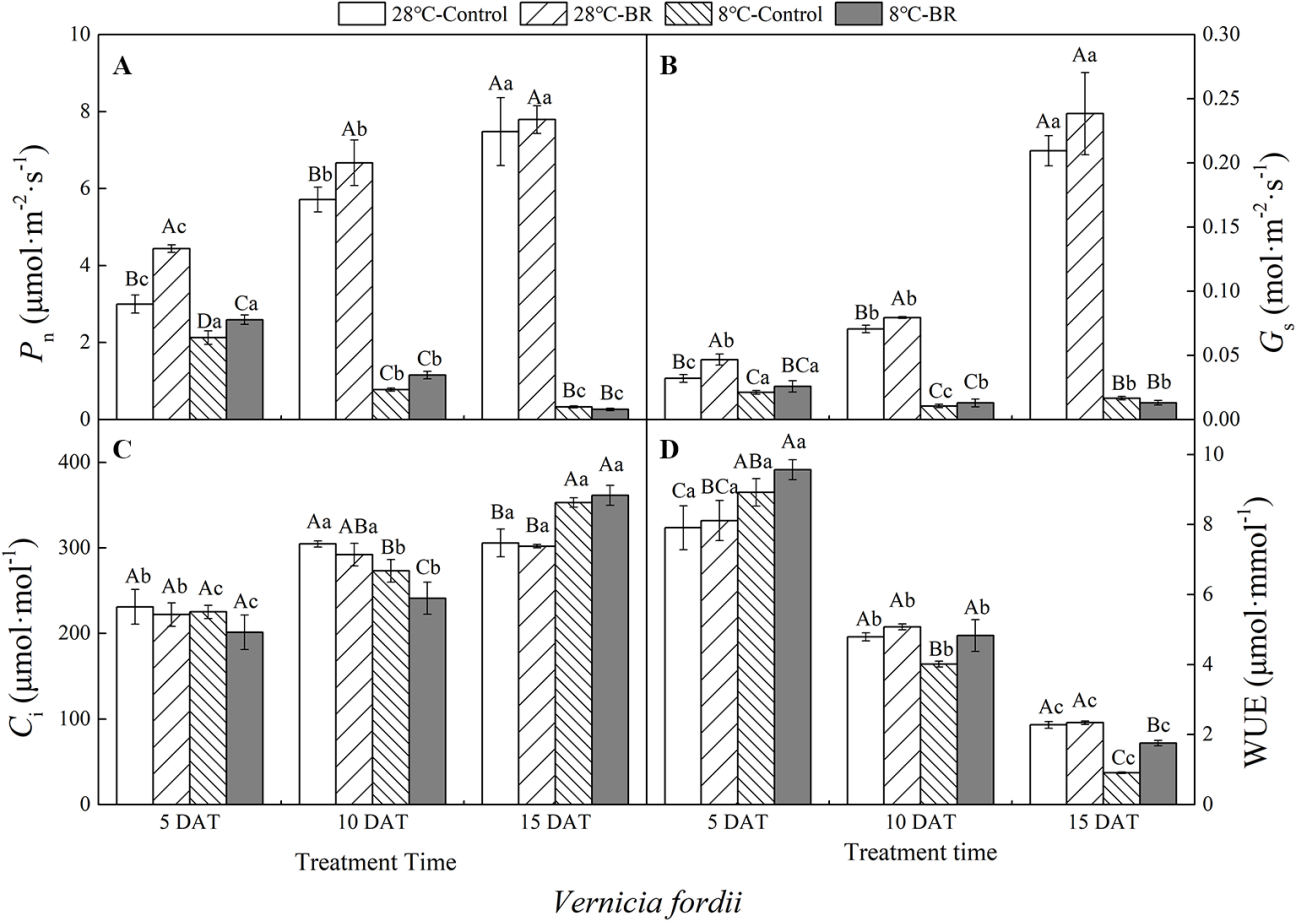
Effects of BR on photosynthetic parameters of *Vernicia fordii* under normal and low-temperature conditions. Treatments were **(A)** 28°C-Control, normal temperature and no BR; **(B)** 28°C-BR, normal temperature and BR application; **(C)** 8°C-Control, low temperature and no BR; and **(D)** 8°C-BR, low temperature and BR application. Different uppercase/lowercase letters indicate significant differences at different treatments and times, indicated by *p ≤* 0.05 according to *Duncan's multiple range tests, and vertical bars indicate ± SE of mean (n = 3)*.

**Figure 3:**
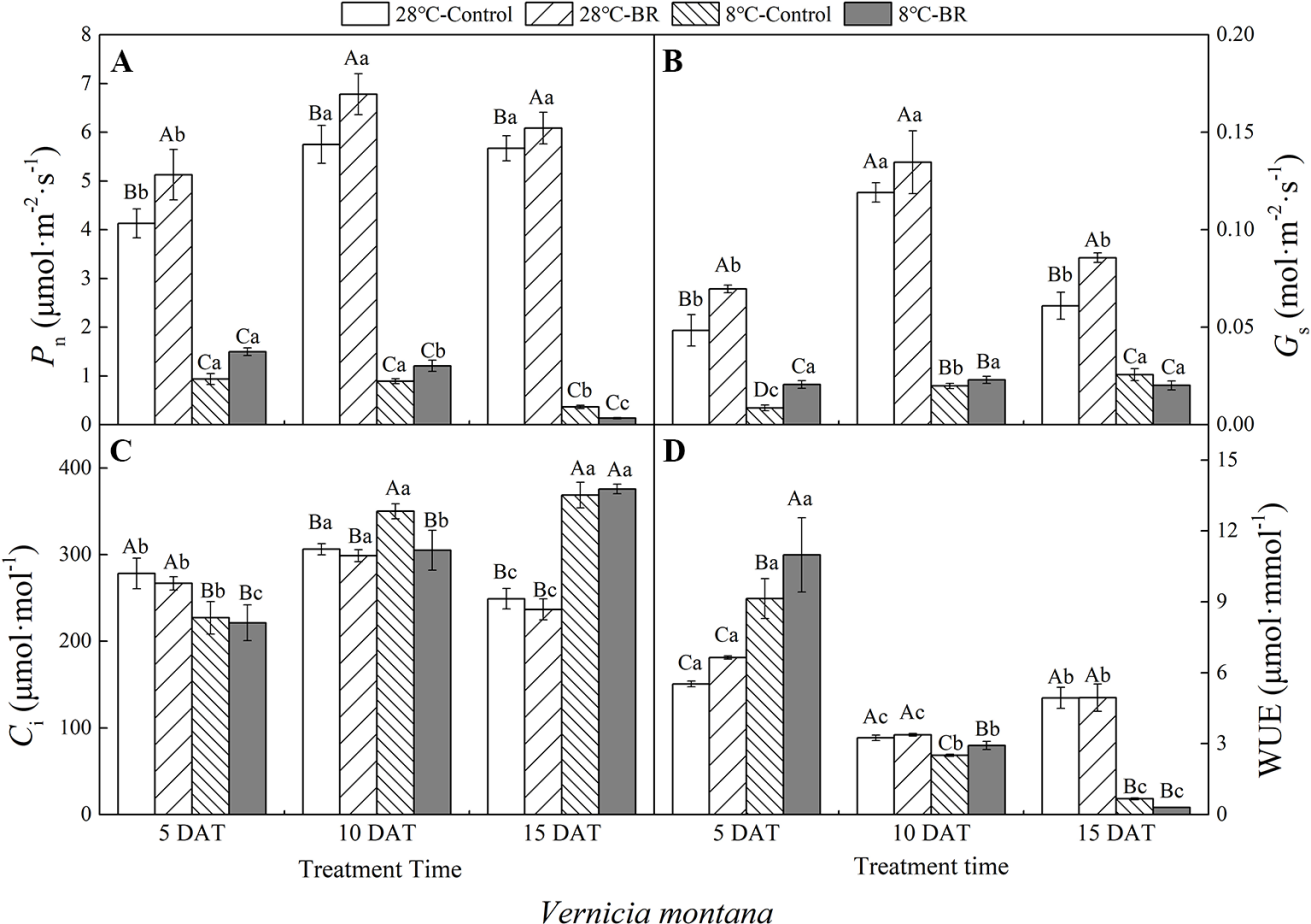
Effects of BR on photosynthetic parameters of *Vernicia montana* under normal and low-temperature conditions. Treatments were **(A)** 28°C-Control, normal temperature and no BR; **(B)** 28°C-BR, normal temperature and BR application; **(C)** 8°C-Control, low temperature and no BR; and **(D)** 8°C-BR, low temperature and BR application. Different uppercase/lowercase letters indicate significant differences at different treatments and times, indicated by *p ≤* 0.05 according to *Duncan's multiple range tests, and vertical bars indicate ± SE of mean (n = 3)*.

The *P_n_*, *G_s_*, and WUE of *V. fordii* significantly decreased as the low-temperature stress continued, whereas the *C_i_* significantly increased (**Figures 2A–C**). However, the *P_n_* and *G_s_* of *V. fordii* always increased under normal-temperature conditions, but the WUE always decreased (**Figures 2A–B**, **D**). The *P_n_* and WUE of *V. montana* significantly decreased as the low-temperature stress continued (**Figures 3A, D**), and the *C_i_* increased significantly (**Figure 3C**). Under low-temperature conditions, the *P_n_* and *G_s_* of *V. montana* leaves were significantly decreased compared to those in normal-temperature conditions (**Figures 3A, B**). The Ci was higher under low-temperature conditions, except at 5 DAT, and the WUE was lower at 10 and 15 DAT (**Figures 3C, D**).

Exogenous spraying of BR increased the *P*_n_, *G*_*s*_, and WUE of both species compared to the control 28°C-Control group. Similar results were observed for both species under low-temperature stress, except for at 15 DAT (**Figures 2** and **3**). Exogenous spraying of BR did not alter this trend but resulted in higher values for each parameter than in non-BR-treated plants at 5 and 10 DAT (**Figures 2** and **3**).

### Chlorophyll Fluorescence Parameters

**Figure 4:**
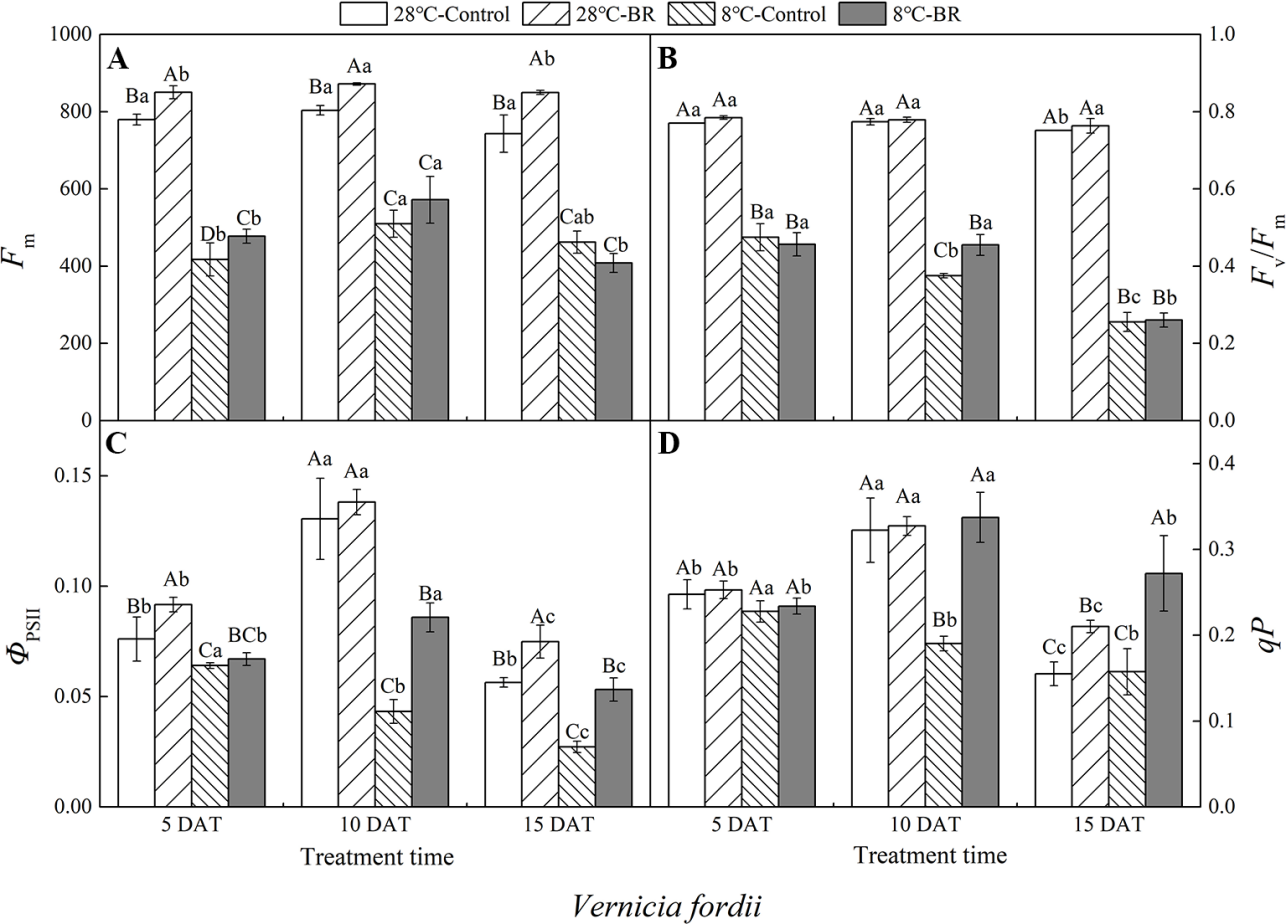
Effects of BR on chlorophyll fluorescence parameters of *Vernicia fordii* under normal and low-temperature conditions. Treatments were (A) 28°C-Control, normal temperature and no BR; (B) 28°C-BR, normal temperature and BR application; (C) 8°C-Control, low temperature and no BR; and (D) 8°C-BR, low temperature and BR application. Different uppercase/lowercase letters indicate significant differences at different treatments and times, indicated by *p* ≤ 0.05 according to *Duncan's multiple range tests, and vertical bars indicate ± SE of mean (n = 3)*.

**Figure 5:**
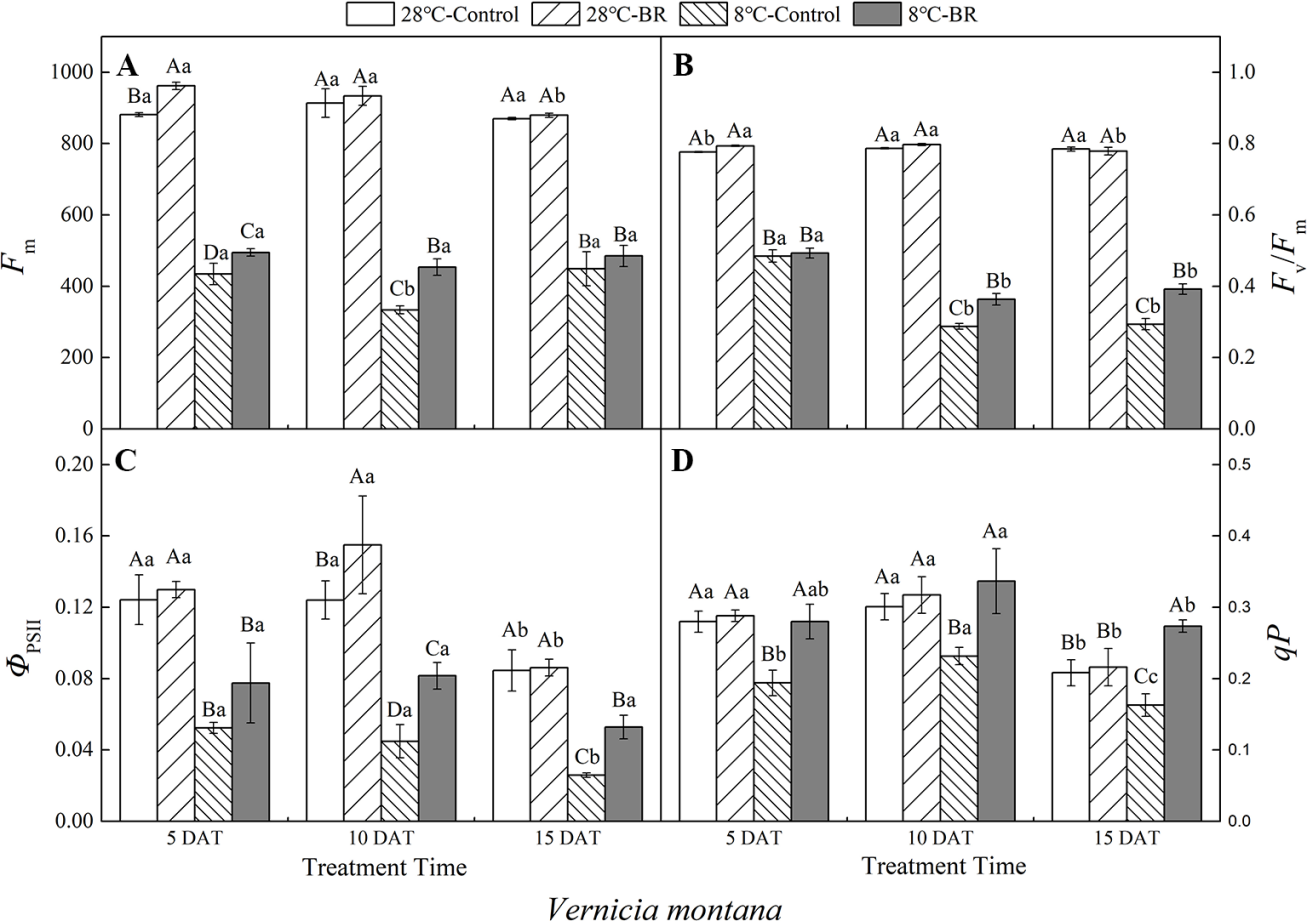
Effects of BR on chlorophyll fluorescence parameters of *Vernicia montana* under normal and low-temperature conditions. Treatments were **(A)** 28°C-Control, normal temperature and no BR; **(B)** 28°C-BR, normal temperature and BR application; **(C)** 8°C-Control, low temperature and no BR; and **(D)** 8°C-BR, low temperature and BR application. Different uppercase/lowercase letters indicate significant differences at different treatments and times, indicated by *p* ≤ 0.05 according to *Duncan's multiple range tests, and vertical bars indicate ± SE of mean (n = 3)*.

Compared with the control 28°C-Control, the *F*_m_, *F*_v_/*F*_m_, *Φ*_PSII_, and *qP* of both species were significantly reduced under low-temperature stress, indicating that a low-temperature environment significantly affects the activity of the photosynthetic apparatus in tung tree seedlings (**Figures 4** and **5**). Exogenous spraying of BR increased the *F*_m_, *F*_v_/*F*_m_, *Φ*_PSII_, and *qP* in both species at 28°C except for the *F*_v_/*F*_m_ in *V. montana* at 15 DAT. These results indicate that the addition of BR did not significantly promote the activity of the photosynthetic apparatus in tung trees under normal-temperature conditions. However, in the 8°C-Control treatment, exogenous spraying of BR resulted in increased *F*_m_ in both tung tree species during the entire experimental period, except for 15 DAT in *V. fordii*, and the *F*_v_/*F*_m_ values were also increased, except for 5 DAT in *V. fordii* (**Figures 4A–B** and **5A–B**). These results matched our expectations.

Under the long-term low-temperature stress of the 8°C-Control treatment, the *F_v_/F_m_*, *Φ*_PSII_, and *qP* showed a continuous downward trend (*p* < 0.05) in *V. fordii* (**Figures 4B–D**). Application of BR had no significant effect on the *Φ*_PSII_ or *qP* at 5 DAT, whereas they increased by 98.41 and 77.22%, respectively, at 10 DAT compared to the 8°C-Control. The *Φ*_PSII_ and *qP* increased by 95.77 and 72.74%, respectively, compared to the control 8°C-Control at 15 DAT, indicating that BR treatment could improve the photosynthetic activity of the tung tree under long-term low-temperature stress conditions (**Figures 4C, D**).

Under long-term low-temperature stress, the *qP* in the 8°C-Control and BR treatment groups increased initially and then decreased (*p* < 0.05) over all three time periods in *V. montana* (**Figure 5D**). The *Φ*_PSII_ in the 8°C-Control treatment showed a significant and continuous downward trend as the low-temperature stress persisted (*p* < 0.05) (**Figure 5C**). However, compared with the 8°C-Control, the *Φ*_PSII_ in the 8°C-BR treatment group improved by 47.99, 81.89, and 104.35% at 5, 10, and 15 DAT, respectively (**Figure 5C**). In addition, the *F_v_/F_m_* under both treatments showed a downward trend as the low-temperature stress persisted, whereas, compared with the control 8°C-Control, the *F_v_/F_m_* of *V. montana* pre-treated with BR increased by 26.48 and 33.55% (*p* < 0.05) at 10 and 15 DAT, respectively (**Figure 5B**).

### Leaf Anatomical Structure

Under low-temperature treatment, the palisade tissue thickness, spongy tissue thickness, and leaf thickness of both species pre-treated with BR were all significantly thicker than those of the control 8°C-Control during the entire experiment, expect for *V. montana* at 15 DAT (**Table 2**). Compared with the 8°C-Control treatment, the leaf thickness of *V. fordii* pre-treated with BR increased by 49.50, 11.56, and 25.58% (*p* < 0.05) at 5, 10, and 15 DAT, respectively. Compared with the 8°C-Control treatment, the CTR of both species pre-treated with BR was higher at 10 and 15 DAT, whereas the SR was lower during the entire experiment (**Table 2**). Compared with the 28°C-Control treatment, the CTR of the 28°C-BR treatment was higher in both species at all time points (**Table 2**), which showed that exogenous spraying of BR increased the proportion of palisade tissue in tung tree leaves. Moreover, exogenous spraying of BR on both species increased the palisade tissue thickness under normal temperatures, consistent with our findings under low-temperature stress. Interestingly, even though exogenous application of BR did not increase the leaf thickness of either species under normal temperatures, external application of BR did increase the leaf thickness under low-temperature stress at 5 and 10 DAT (**Table 2**).

**Table 2:** Effects of BR on leaf anatomical structure parameters of two tung tree species under normal and low-temperature conditions.

*Species*	Treatment time/DAT	Treatment	Palisade tissue thickness (µm)	Spongy tissue thickness (µm)	Leaf thickness (µm)	CTR (%)	SR (%)
*Vernicia fordii*	5	8°C-Control	52.04 ± 0.81Ca	72.86 ± 1.57Cb	154.13 ± 0.85Da	33.77 ± 0.70Aa	47.27 ± 0.78Bb
		8°C-BR	69.06 ± 2.62Aa	105.85 ± 5.30Aa	230.43 ± 2.74Aa	29.97 ± 1.12Bc	45.92 ± 1.78Bb
		28°C-Control	49.56 ± 1.10Ca	104.39 ± 2.75Aa	184.55 ± 1.60Ba	26.86 ± 0.80Cc	56.56 ± 1.06Aa
		28°C-BR	62.03 ± 3.06Ba	85.44 ± 4.78Ba	178.10 ± 1.12Ca	34.84 ± 1.93Ab	47.98 ± 2.75Bab
	10	8°C-Control	44.64 ± 0.74Bb	66.55 ± 1.67Bc	136.06 ± 2.61Bc	32.81 ± 0.75Ca	48.93 ± 1.79Ab
		8°C-BR	60.52 ± 2.40Ab	73.67 ± 2.26Ac	151.79 ± 2.95Ac	39.87 ± 0.97Aa	48.53 ± 0.93ABb
		28°C-Control	44.04 ± 0.76Bb	53.25 ± 0.41Cc	116.89 ± 3.29Cc	37.71 ± 1.71Ba	45.58 ± 1.54BCc
		28°C-BR	46.82 ± 0.73Bb	50.29 ± 2.71Cc	112.00 ± 3.58Cc	41.79 ± 1.12Aa	44.88 ± 1.15Cb
	15	8°C-Control	35.21 ± 1.86Dc	82.28 ± 1.42Ba	145.81 ± 2.11Bb	24.16 ± 1.61Cb	56.43 ± 1.09Aa
		8°C-BR	59.56 ± 1.67Ab	95.14 ± 2.22Ab	183.12 ± 1.47Ab	32.53 ± 1.00Bb	51.96 ± 1.54Ba
		28°C-Control	39.90 ± 0.96Cc	68.26 ± 2.26Cb	126.41 ± 1.80Cb	31.57 ± 0.91Bb	53.99 ± 1.18ABb
		28°C-BR	42.84 ± 0.73Bb	64.14 ± 2.23Db	120.66 ± 5.34Cb	35.56 ± 1.93Ab	53.28 ± 4.12ABa
*Vernicia montana*	5	8°C-Control	58.59 ± 0.20Cb	53.44 ± 0.33Cb	131.64 ± 1.40Db	44.51 ± 0.38BCa	40.60 ± 0.43Aa
		8°C-BR	75.72 ± 1.27Aa	66.81 ± 1.38Aa	162.89 ± 3.39Aa	46.50 ± 1.10Aa	41.04 ± 1.67Aa
		28°C-Control	58.60 ± 1.05Ca	53.58 ± 1.61Cb	135.66 ± 1.33Cb	43.20 ± 1.05Ca	39.50 ± 1.57Aa
		28°C-BR	69.49 ± 0.69Ba	62.18 ± 0.77Ba	154.71 ± 0.54Ba	44.92 ± 0.39ABa	40.19 ± 0.36Aa
	10	8°C-Control	50.49 ± 1.28Cc	47.81 ± 5.22Bb	120.59 ± 5.69Bc	41.92 ± 1.83Ba	39.73 ± 4.94Aa
		8°C-BR	61.73 ± 0.94Bb	55.26 ± 2.12Ab	140.15 ± 1.23Ab	44.05 ± 1.01ABb	39.42 ± 1.25Aa
		28°C-Control	61.78 ± 2.85Ba	57.02 ± 2.45Aab	144.13 ± 2.20Aa	42.87 ± 2.15Ba	39.56 ± 1.61Aa
		28°C-BR	67.10 ± 0.43Aab	48.07 ± 0.49Bc	145.55 ± 0.74Ab	46.10 ± 0.44Aa	33.03 ± 0.34Bc
	15	8°C-Control	63.39 ± 3.29Aa	61.16 ± 2.08Aa	151.86 ± 1.85Aa	41.75 ± 2.33Aa	40.27 ± 0.99ABa
		8°C-BR	60.15 ± 1.20Ab	55.09 ± 3.23BCb	140.00 ± 1.26Cb	42.97 ± 0.75Ab	39.35 ± 2.24ABa
		28°C-Control	60.93 ± 1.95Aa	59.25 ± 1.43ABa	144.92 ± 4.27Ba	42.08 ± 2.37Aa	40.93 ± 2.21Aa
		28°C-BR	65.11 ± 3.27Ab	52.77 ± 2.21Bb	142.89 ± 0.37BCc	45.57 ± 2.32Aa	36.93 ± 1.62Bb

Data represent mean values ± standard error (SE) (n = 3). Treatments were (1) 28°C-Control, normal temperature and no BR; (2) 28°C-BR, normal temperature and BR application; (3) 8°C-Control, low temperature and no BR; and (4) 8°C-BR, low temperature and BR application. Different uppercase/lowercase letters indicate significant differences at different treatments and times, indicated by p ≤ 0.05 according to *Duncan's multiple range tests. CTR is cell tense ratio and SR is spongy ratio*.

**Figure 6:**
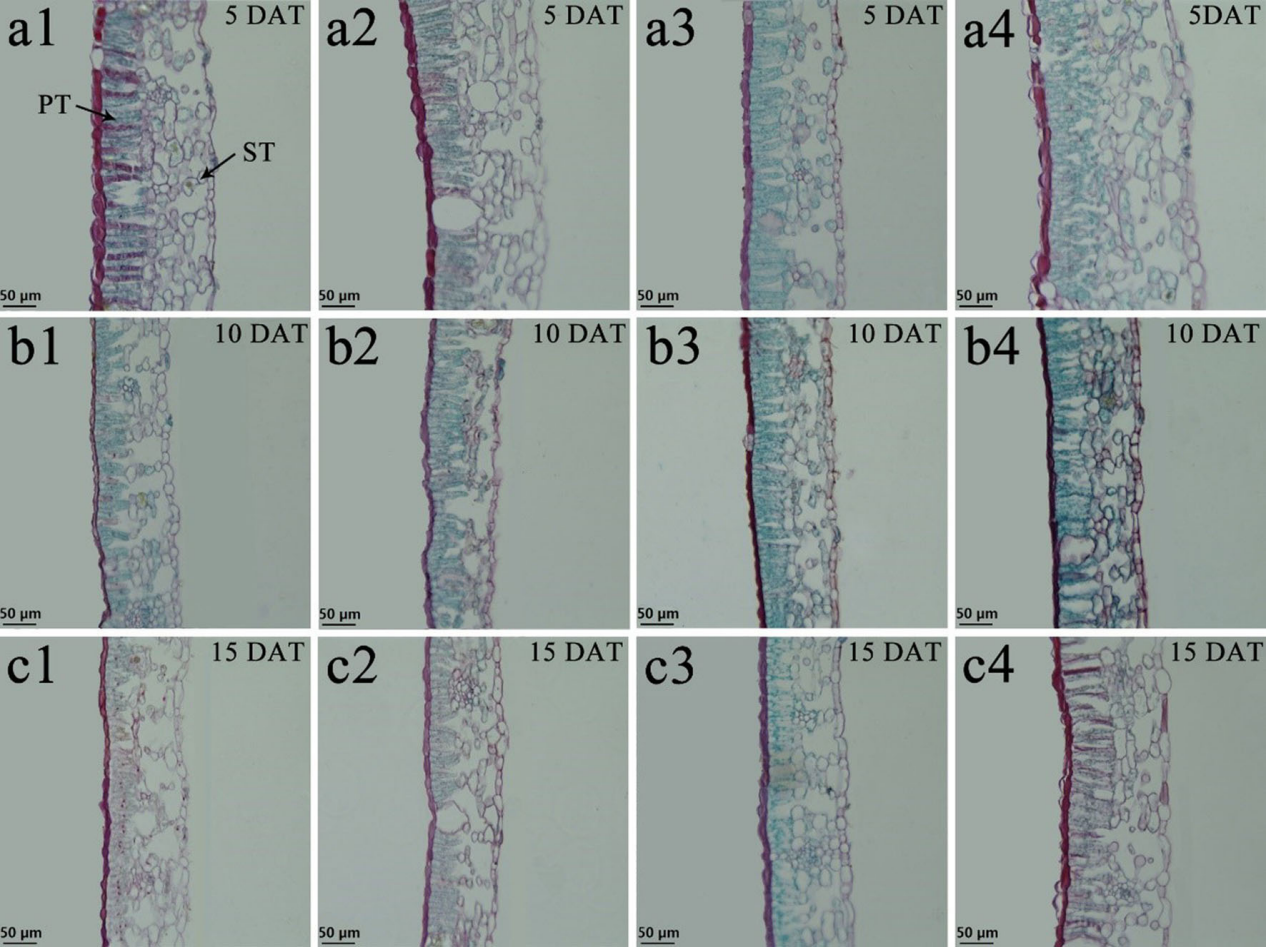
Effects of BR on leaf anatomical structure of *Vernicia fordii* under normal and low-temperature conditions. Treatments were (1) 28°C-Control, normal temperature and no BR, a1-c1; (2) 28°C-BR, normal temperature and BR application, a2–c2; (3) 8°C-Control, low temperature and no BR, a3–c3; and (4) 8°C-BR, low temperature and BR application, a4-c4. All the microscopic multiples were 20× ×DAT, days after treatment; PT, palisade tissue; ST, spongy tissue. Bars, 50 μm.

**Figure 7:**
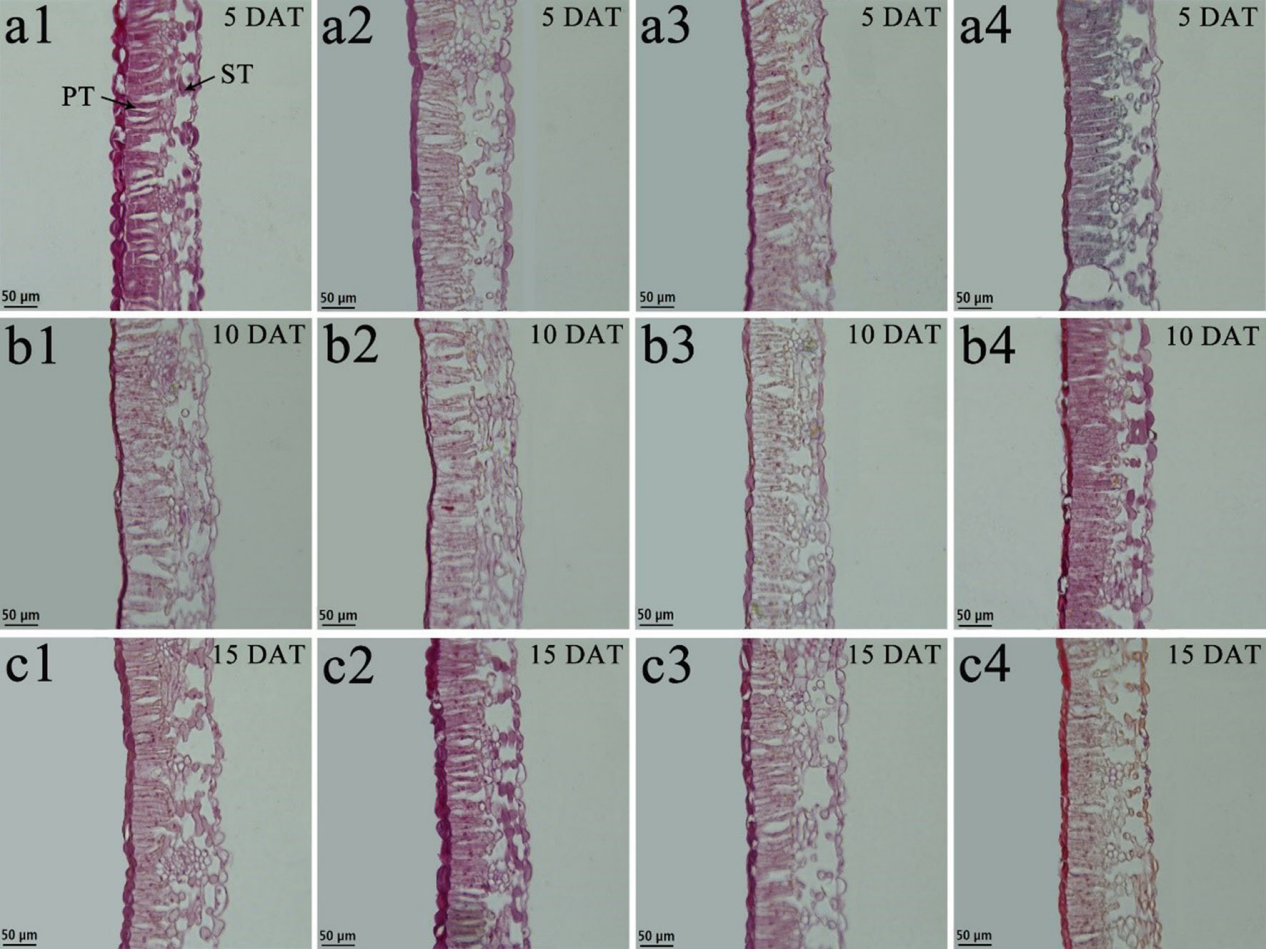
Effects of BR on leaf anatomical structure of *Vernicia montana* under normal and low-temperature conditions. Treatments were (1) 28°C-Control, normal temperature and no BR, a1-c1; (2) 28°C-BR, normal temperature and BR application, a2–c2; (3) 8°C-Control, low temperature and no BR, a3–c3; and (4) 8°C-BR, low temperature and BR application, a4-c4. All the microscopic multiples were 20× ×. DAT, days after treatment; PT, palisade tissue; ST, spongy tissue. Bars, 50 μm.

Tung tree leaves are composed of the upper epidermis, lower epidermis, and mesophyll. The epidermis consists of irregular oblong monolayers of varying sized cells, and the mesophyll consists of a layer of palisade tissue cells and multiple layers of spongy tissue cells (**Figures 6** and **7**). Under normal-temperature conditions, exogenous spraying of BR did not significantly alter the leaf anatomical structure of either species compared with the control 28°C-Control (**Figures 6a1–c1, 6a2–c2**, **7a1–c1**, and **7a2–c2**). Compared with the 28°C-Control treatment, the palisade tissue of *V. fordii* and the spongy tissue of *V. montana* were loosened and had increased cell spacing under low-temperature stress at 5 DAT (**Figures 6a3** and **7a3**). The palisade tissue of both species and the spongy tissue of *V. montana* were loosened and the cell spacing larger at 15 DAT (**Figures 6c3** and **7c3**), but exogenous application of BR prevented this effect (**Figures 6**–**7a4** and **6**–**7c4**).

### Chloroplast Ultrastructure Observation

**Figure 8:**
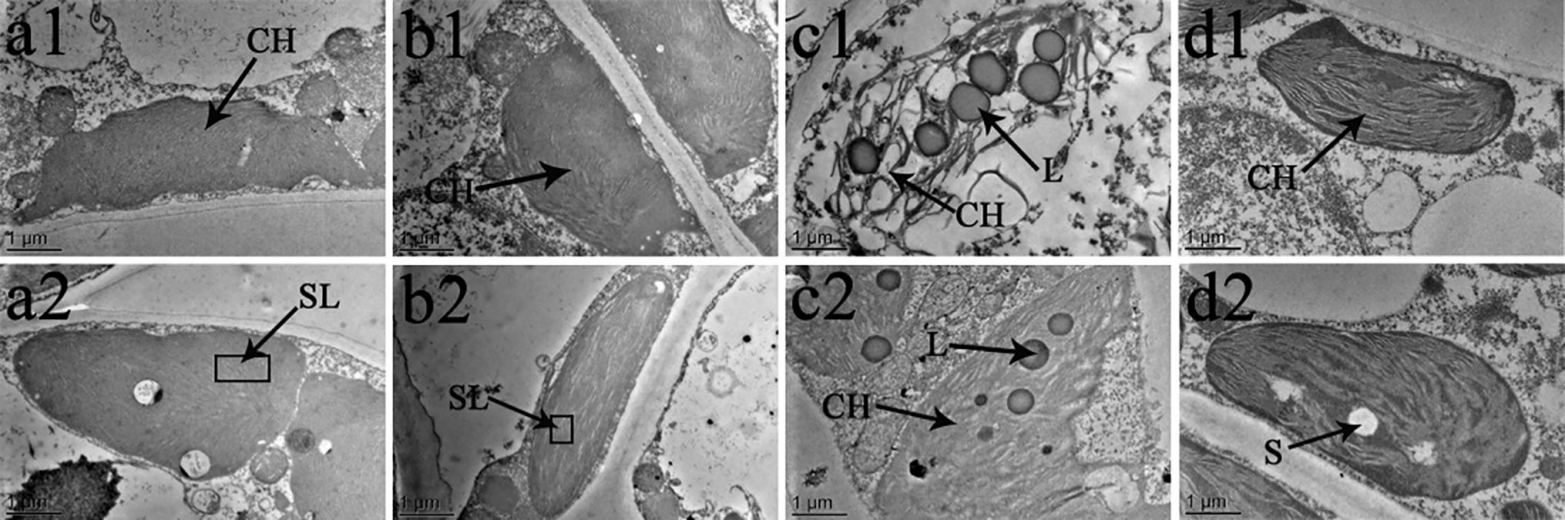
effects of br on chloroplast ultrastructure of *vernicia fordii* under normal and low-temperature conditions. Treatments were (1) 28°C-Control, normal temperature and no BR, a1–2; (2) 28°C-BR, normal temperature and BR application, b1–2; (3) 8°C-Control, low temperature and no BR, c1–2; and (4) 8°C-BR, low temperature and BR application, d1-2. CH, chloroplast; SL, stroma lamella; L, liposomes; S, starch. Images were photographed with TEM.

**Figure 9:**
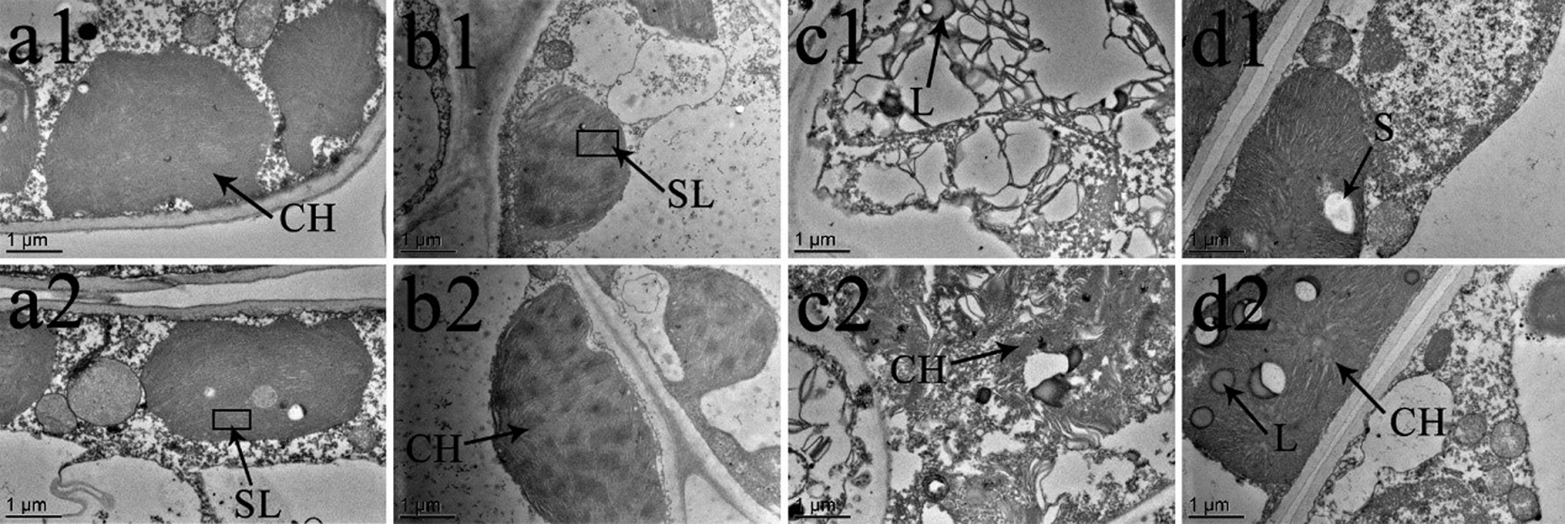
Effects of BR on chloroplast ultrastructure of *Vernicia montana* under normal and low-temperature conditions. Treatments were (1) 28°C-Control, normal temperature and no BR, a1–2; (2) 28°C-BR, normal temperature and BR application, b1–2; (3) 8°C-Control, low temperature and no BR, c1–2; and (4) 8°C-BR, low temperature and BR application, d1–2. Abbreviations: CH, chloroplast; SL, stroma lamella; L, liposomes; S, starch. Images were photographed with TEM.

The chloroplast ultrastructure of both species changed under the different temperature and BR treatments, as shown in **Figures 8** and **9**. Chloroplasts from samples grown under normal temperature conditions were almost adjacent to the cell wall, and the cell shapes were mainly regular and integral (**Figures 8a1, b1, a2, b2**, **9a1, b1, a2, b2**). Moreover, few starch granules were observed in most chloroplasts, and the stroma lamellae were neatly arranged (**Figures 8A–B** and **9A–B**). In contrast, the stroma lamellae of the 28°C-BR *V. fordii* were slightly expanded (**Figure 8B**). Few chloroplasts of the 8°C-Control treatment in *V. fordii* had a normal appearance, and some of their structures were destroyed. In addition, most of the chloroplast structure in *V. montana* was destroyed and the thylakoid structure was loosened and disintegrated (**Figure 9C**). Moreover, the number of liposomes in the chloroplasts was increased in *V. montana* (**Figures 8C**, **9C**). However, the chloroplasts in both species pre-treated with BR remained intact (**Figures 8D** and **9D**). In both species, the chloroplasts contained a small amount of starch, and the stroma lamellae were loosely arranged, with slight swelling and expansion (**Figures 8D** and **9D**). Also, the number of liposomes in *V. montana* chloroplasts was increased (**Figure 9D**).

## Discussion

Plant photosynthetic processes are highly sensitive to environmental changes and depend on aspects such as chlorophyll content, photosynthetic parameters, and chlorophyll fluorescence parameters (Stirbet and Govindje, 2011). Low-temperature stress increases reactive oxygen species (ROS) in plant metabolic pathways, reduces tolerance to antioxidant enzymes, damages protein and DNA, accelerates chlorophyll decomposition in leaves, slows down plant metabolism, reduces the availability of chlorophyll synthesis substrate, and, thus, results in a reduced leaf chlorophyll content (Yang et al., 2016; Liu et al., 2019b). In our study, with prolonged low-temperature stress, the Chl a, Chl b, and Chla+b contents of both species continued to decrease and were lower than those of the plants maintained under normal temperature conditions, as reported previously. However, our chlorophyll content results at 5 DAT under low-temperature conditions were higher than those under normal temperatures, in contrast to previous findings (Yang et al., 2016). This might be due to the changes in endogenous hormone levels, information transmission, and the regulation of plant physiological responses in both species under short-term low-temperature stress (Kurepin et al., 2015). Plants are able to construct a low-temperature defense system that actively increases their chlorophyll contents and prevents decreases in photosynthesis and energy production caused by a gradual decrease in the chlorophyll content. In contrast, plants maintained at the correct temperature do not need to increase their rate of photosynthesis and energy production by increasing their chlorophyll content. Exogenous application of BR to cotton seedlings under low-temperature stress increased the Chl a, Chl b, and Chla+b contents and protected chloroplasts (Li et al., 2018), which is similar to our results. However, the Chl a, Chl b, and Chl a+b contents in the control-treated plants were higher than those in the BR-treated plants at 15 DAT under low-temperature conditions. It is possible that an appropriate concentration of BR promotes chlorophyll synthesis, whereas a high concentration of BR inhibits the activities of chlorophyll enzymes (Hayat et al., 2010). In addition, exogenous hormone application has been shown to promote the production of endogenous hormones in plants (Muhammad et al., 2006). Therefore, under long-term low-temperature stress, high levels of endogenous BR may accumulate, leading to an inhibition of the activities of chlorophyll enzymes and reduction chlorophyll synthesis in the leaves of both species.

Photosynthesis requires the balancing of the light energy absorbed and the energy consumed by the plant's metabolic sinks and is therefore very sensitive to environmental changes. Low temperature exacerbates the imbalance between the energy source and the metabolic sink, causing significant changes in photosynthesis (Ensminger et al., 2006). In this study, the *P*_n_ of both species of tung tree seedlings grown in low-temperature conditions decreased significantly and continued to decline as the period of stress prolonged, seriously affecting the normal growth of the plants. Studies have shown that the decreased photosynthesis caused by the low-temperature stress is due to both stomatal and non-stomatal factors (Allen and Ort, 2001; Yamori et al., 2012; Cao et al., 2017). When *C_i_* and *G_s_* decrease simultaneously, the decrease in *P_n_* is mainly caused by stomatal factors. When *P_n_* decreases along with an increase in *C_i_*, the main limiting factors of photosynthesis are non-stomatal factors (Farquhar and Sharkey, 1982). In this study, the decrease in *P*_n_ in both species was accompanied by a simultaneous decrease in *C*_i_ and *G*_s_, indicating that stomatal factors were the main limiting factors for photosynthesis at 5 DAT. At 10 DAT, the *P*_n_ of *V. fordii* decreased with a simultaneous decrease in *C*_i_ and *G*_s_. Compared with the initial stages of stress, the *G*_s_ was lower, while the *C*_i_ began to increase. The *P*_n_ of *V. montana* decreased with the increase in *C*_i_, whereas the *G*_s_ was slightly higher than that at the initial stage of stress. This indicates that the limiting factors for photosynthesis in both species during the middle period of stress included both stomatal and non-stomatal factors. The stomatal factors were the dominant limiting factors for photosynthesis in *V. fordii*, but non-stomatal factors were the dominant limiting factors for *V. montana*. As the stress period lengthened, the *P*_n_ of both species continued to decrease, whereas the *C*_i_ and *G*_s_ kept increasing. This indicates that non-stomatal factors were the main limiting factors for photosynthesis at 15 DAT. Previous studies have shown that exogenous BR treatment improved the apparent quantum efficiency, dark respiration rate, and carboxylation efficiency of photosynthetic enzymes, thereby increasing the net photosynthetic rate of plants and increasing the accumulation of organic matter (Yu et al., 2002; Anuradha and Rao, 2009; Li et al., 2017b). Similarly, in our study, the *P*_n_ with BR treatment was higher than that in the control for both species under normal temperatures and at the early and middle stages of cold stress, indicating that exogenous BR treatment increases the photosynthetic rate and cold resistance and alleviates the decrease in *P*_n_ caused by cold. However, the *P*_n_ with BR treatment under cold conditions was lower than that in the control at 15 DAT, which may be due to the increases in the endogenous BR synthesis induced by long-term low-temperature stress. Excessive accumulation of endogenous BR leads to a decrease in the *P*_n_ (Muhammad et al., 2006; Hayat et al., 2010). However, the reduced *P*_n_ may lead to an increased WUE, thus protecting the activity of the photosynthetic apparatus.

Chlorophyll fluorescence parameters could be used to evaluate the function of the photosynthetic apparatus and the effects of low-temperature stress on plants. These parameters could reflect the photosynthetic potential of plants, the ability of plants to convert light energy into chemical energy, and the photosynthetic activity level (Schreiber et al., 1986; Demmig-Adams, 1990; Olaf and Jan, 1990; Wolfgang and Olle, 1990). This study showed that low-temperature stress reduced the *F*_m_ in both tung tree species. The lower *F*_m_ means that the non-photochemical processes of the photosynthetic organs increased, and the photoelectron transfer efficiency and potential decreased (Li et al., 2017b). Moreover, the *F*_*v*_/ *F*_*m*_, *Φ*_*PSII*_, and *qP* decreased in both species during low-temperature stress, indicating that the photosynthetic potential, photosynthetic activity, the ability to convert light energy into chemical energy, and the ability to dissipate excess light energy into heat decreased to varying degrees. Thus, cold conditions increased the degree of leaf inhibition. Exogenous application of BR increased the *F*_m_, maintained the activity of the PSII reaction center, shortened the non-photochemical processes of the photosynthetic organs, and enhanced the efficiency and potential of photoelectron transfer in both species during the low-temperature stress. At the same time, the decreases in *F*_*v*_/ *F*_*m*_ and *Φ*_*PSII*_ were accompanied by decreases in *P*_n_, *G*_s_, and *T*_r_, indicating that the non-stomatal factors responsible for the decreased photosynthetic rate in both species during the middle and late stages of low-temperature stress were due to the reduced activity of the photosynthetic apparatus. As the stress time extended, the *F*_*v*_ */F*_*m*_ and *Φ*_*PSII*_ of *V. fordii* and *V. montana* subjected to control treatment continued to decrease, indicating that the degree of damage to the photosynthetic organs increased with the longer stress time. The *F*_*v*_ */F*_*m*_, *Φ*_*PSII*_, and *qP* of the BR-treated plants were always higher than that in control-treated plants, indicating that exogenous BR spraying alleviated the damage to the photosynthetic organs of the tung tree seedlings during low-temperature stress and promoted photosynthesis. Clearly, our results from the present study are consistent with the other early reports (Anuradha and Rao, 2009; Yu et al., 2015).

Anatomical observation of the plant tissues is important to clarify any internal changes. Leaves are sensitive to low-temperature stress as they are the main organs for photosynthesis and transpiration. Therefore, changes in the leaf anatomical structure are the basis of the plant response and adaptation to environmental changes (Schreiber et al., 2013; Kaur et al., 2019). Leaf structural parameters were affected by the environment but did not accurately indicate the cold resistance of the plants. CTR and SR could be used to measure the cold resistance of plants, as they combine the differences in palisade tissue thickness, spongy tissue thickness, and total leaf thickness. A higher CTR and lower SR indicate increased cold resistance (Cao et al., 2014). The CTRs of the two tung tree species under low-temperature conditions were lower than that under normal temperatures, and the SRs were higher than that under normal temperatures during the middle and late stages of stress, suggesting that a long term low temperature treatments can affect the leaf structure and compactness. However, the results are in contrast to those at the early stage of the low temperature stress. This may be due to the fact that the plants that were exposed to cold conditions, in order to prevent the damage to the structure of leaves caused by a low-temperature environment, improved their CTR and enhanced leaf tightness. Moreover, in this study, with the prolongation of low-temperature stress, the CTR in *V. fordii* continued to decline, but the SR continued to increase, indicating that the long-term low-temperature stress seriously affected the internal structure of leaves, weakened the cold resistance potential of plants, and aggravated the damage to the plant. However, the changes in the CTR and SR in *V. montana* were less apparent than that in *V. fordii*. This might be due to the *V. montana* leaf tissues which are capable of adapting to low temperature conditions by increasing or decreasing the thickness of the palisade tissue and spongy tissue at the same time, so that the tightness and looseness of the leaf tissue were relatively consistent (Teng et al., 2018). At the low temperatures conditions, the CTR following BR treatment was always higher than that following the control (except for *V. fordii* at 5 DAT), and the SR following BR treatment (except for *V. montana* at 5 DAT) was lower than that following control, suggesting that the exogenous application of BR improved the cold resistance of plants and promoted the stability of the leaf structure.

As the photosynthetic organs, chloroplasts are vulnerable to stress-induced damages. Early structural changes occur in the chloroplasts when the leaves suffer from abiotic stresses (Li et al., 2018). Therefore, examination of the chloroplast ultrastructure is important to investigate photosynthesis and the effects of abiotic stress on the photosynthetic apparatus (Hu et al., 2015). In this study, we compared the changes in the chloroplast ultrastructure of two tung tree species at 15 DAT. We found that a low-temperature treatment significantly damaged the chloroplast structure, loosening the thylakoids until they were disintegrated and increasing the number of liposomes in the chloroplasts, indicating that the photosynthetic apparatus was destroyed in both species during the late stage of the low-temperature stress. Similar observations have also been made by other workers (Taylor and Craig, 1971; Marzanna et al., 2002). Thus, we can conclude that the decrease in the rate of photosynthesis during the late stage of low-temperature stress was mostly due to non-stomatal factors that damaged the photosynthetic mechanism by analysing results above. Furthermore, most of the chloroplasts in *V. montana* leaves had disintegrated, and the damage was even more severe than that in *V. fordii*. In contrast to the control, the chloroplasts of plants sprayed with BR were normal and intact, and only the stroma lamellae were loosely arranged and slightly swollen and expanded. In addition, a small number of starch granules were present in the chloroplasts, and the number of liposomes in the *V. montana* chloroplasts was increased. This finding is consistent with those of Yuan et al. (2012) and Gill et al. (2017) and suggests that exogenous application of BR can protect the chloroplasts of both tung tree species, alleviating the damage to the photosynthetic apparatus caused by long-term low-temperature treatments, maintaining photosynthetic efficiency, and ensuring the basic survival needs of the plants. This supported the above conclusion that long-term low temperature conditions could limit the tung tree growth by damaging the chloroplasts structure and reducing the photosynthetic product production, while exogenous application of BR could increase the activity of photosynthetic apparatus, which might be a potential mechanism for tung trees to tolerate low-temperature stress through investing more energy into protecting the plant rather than having the energy produced by chloroplasts be consumed on the chloroplasts when there is no externally applied BR. Therefore, we could conclude that exogenous application of BR could be a potential way to prevent tung tree to suffer from the damage of low temperature stress.

Comparison of the photosynthetic characteristics between *V. fordii* and *V. montana* under the low-temperature conditions has revealed that damage to the photosynthetic mechanism in *V. montana* is the main factor affecting photosynthesis in the middle period of stress, whereas the damage to *V. fordii* does not become a dominant factor until the late period of stress. In addition, based on observations of the chloroplast ultrastructure, we have found that the low temperature treatment causes less damage to the chloroplasts in *V. fordii* than in *V. montana*. Therefore, it can be concluded that *V. fordii* has greater cold resistance than *V. montana*.

## Conclusions

Our results clearly showed that low temperature caused decreases in the *P_n_* and WUE, slowed growth, reduced photosynthesis, altered the internal leaf structure, and destroyed the chloroplast structure thus negatively affecting the normal growth and development of *V. fordii* and *V. montana* seedlings. Exogenous spraying of BR enhanced the photosynthetic potential, shortened the non-photochemical processes of the photosynthetic organs, increased the efficiency and potential of photoelectron transmission, maintained the activity of the photosynthetic organs, promoted the stability of the leaf structure and morphology, and effectively alleviated the damage caused by the cold stress. Moreover, both stomatal and non-stomatal factors played an important role in the reduction of the photosynthetic efficiency of tung tree seedlings caused by the cold stress. The decrease in the photosynthetic efficiency caused by the short-term cold-induced injury was mainly due to stomatal factors, while the decrease in photosynthetic efficiency caused by the long-term cold-induced injury was mainly caused by non-stomatal factors that damaged the photosynthetic mechanism. Finally, *V. fordii* exhibited a stronger cold resistance to cold condition than *V. montana*.

## Data Availability Statement

All datasets generated for this study are included in the article/supplementary material.

## Author Contributions

FZ analyzed the results. KL and YG prepared plant materials and collected the samples. LZ and WL prepared **Figures 6**–**9**. FZ, KL, and ZL wrote the main manuscript text. All authors reviewed the manuscript.

## Conflict of Interest

The authors declare that the research was conducted in the absence of any commercial or financial relationships that could be construed as a potential conflict of interest.
